# Supplementary material for: Economic damages from Hurricane Sandy attributable to sea level rise caused by anthropogenic climate change
Source: Nat Commun. 2021 May 18;12:2720. doi: 10.1038/s41467-021-22838-1 (PMC8131618; doi:10.1038/s41467-021-22838-1)
Supplement: Supplementary file 1 — Supplementary Information [file 41467_2021_22838_MOESM1_ESM.pdf]

Supplementary Information: Economic Damages from Hurricane Sandy Attributable to Sea Level Rise Caused by Anthropogenic Climate Change

Benjamin Strauss[1], Philip Orton[2], Klaus Bittermann[3,4], Maya K. Buchanan[1], Daniel M. Gilford[1,5], Robert E. Kopp[5], Scott Kulp[1], Chris Massey[6], Hans de Moel[7], and Sergey Vinogradov[2,8]

[1] Climate Central, Princeton, NJ, USA bstrauss@climatecentral.org,
[2] Stevens Institute of Technology, Hoboken, NJ, USA
[3] Tufts University, Boston, MA, USA
[4] Potsdam Institute for Climate Impact Research, Potsdam, Germany
[5] Department of Earth & Planetary Sciences and Rutgers Institute of Earth, Ocean, and Atmospheric Sciences, Rutgers University, New Brunswick, NJ, USA
[6] USACE, Washington, DC, USA
[7] Vrije Universiteit Amsterdam, The Netherlands
[8] NOAA, Silver Spring, MD, USA

1 Supplementary Methods

1.1 Flood modeling

The coupled ADCIRC/STWAVE model used here is one of the best available coastal flood models for a simulation over such a large area (the "Sandy-affected area"), balancing speed, resolution and achieving high accuracy. ADCIRC is a very widely-used model for coastal flood studies, including increasing adoption by the Corps of Engineers (e.g. the North Atlantic Coast Comprehensive Study) and by NOAA for real-time forecasting (https://ocean.weather.gov/estofs/estofs_surge_info.php) and post-disaster hurricane flood hindcasts (https://www.weather.gov/sti/coastalact_surgewg).

An important concern with hydrodynamic flood modeling is adequate resolution to capture any flood defenses, which can be linear features of small scale. The ADCIRC mesh used in this study is the same 3.1-million-node mesh used in the North Atlantic Coast Comprehensive Study (NACCS) (1). The portion of the NACCS mesh covering the New York area was expanded, enhanced and updated from a FEMA Region II Flood Risk Map Study (2), where the mesh was specifically designed to have nodes aligning with coastlines and river boundaries and vertical features such as boardwalks, jetties, roadways as well as existing flood defenses such as dunes, berms and floodwalls. In the Sandy-affected areas the mesh resolution was typically 70 m. Elevated vertical features such as shoreline berms have resolutions as low as 50 m and were delineated in mesh creation so as to optimally resolve the elevated spine of the feature. Small-scale geographical features such as tributaries have a resolution as low as 10 m (1). The mesh underwent extensive validation as part of the North Atlantic Coast Comprehensive Study (1) including extratropical storm events that did not produce as much flooding as Sandy. ADCIRC is run on a pre-existing unstructured mesh covering the Northwest Atlantic, including the US East Coast and Gulf of Mexico, with 3.1

million nodes, where the typical Sandy floodplain resolution is 70 m but areas small-scale geographic features such as tributaries have a resolution as low as 10 m.

STWAVE is run with existing grids with resolutions between 100 and 200 m (1) with horizontal resolution in the coastal tri-state area up to 70 m for the unstructured circulation/surge model. Tight coupling between ADCIRC and STWAVE is achieved using the CSTORM coupling framework (3) whereby the models share information via computer memory during runtime in order to update forcing values between the two models. The Hudson River north of Tappan Zee was included in the ADCIRC mesh for correctness of volume of fluid but was not resolved to an extent necessary for accurate modeling in the ADCIRC mesh. As a result, our results do not include Hudson River floodplain areas north of Tappan Zee, which constitute a negligible proportion (below 0.1%) of the Hurricane Sandy damages.

Simulations each cover 20.5 days, starting at 2012 October 11 12:00 GMST using 8 tidal constituents (M2,S2,N2,K1,O1,Q1,P1,K2) and prescribed river inflows. Winds and pressures are turned on after a 13.5 day long spin-up period of tides and rivers, and updated every 15 minutes. Nearshore waves are turned on after 16.5 days and updated every 30 minutes which allows for nearshore wave contributions to the surge to be started early enough to allow for proper influence but not be overly computationally burdensome. We use the default wind drag parameterization for ADCIRC (4), with a saturation ceiling value of 0.0035 (e.g., 1; 5). Manning's n values for areas below mean sea level are set at 0.02, and for upland areas are variable (based on the GAP land-cover database), both as in the recent coastal flood study for this region (5) .

Our hydrodynamic modeling captures the influence of sea level rise on tides within the model domain, including amplification within estuaries (e.g. Long Island Sound; (6)). However, it cannot capture any Atlantic basin-wide changes. At the open coast in the New York Bight region (e.g. Atlantic City, Sandy Hook) the total of these changes is below 1 cm per half-meter of sea level rise (Schindelegger et al. 2018), some fraction of which is captured in our model domain. Therefore, any missing basin-wide changes to tides due to sea level rise are on the order of a few millimeters, negligible for our study's purposes.

The validation using pre-Sandy bathymetry (Supplementary Figure 1) shows RMS errors in maximum water elevation of 0.355 m on peak water elevations of up to 4.2 m. Therefore, the model is capturing the physical processes of storm surge and tide very well. For comparison, a 2019 model study of Hurricane Matthew's coastal storm tides across several states had RMS errors on water level of 0.28 m, relative to a peak water elevation of 2.9 m (7). Considering that the water level errors have strong spatial coherence, we may express the observed maximum water levels as:

$$h_{obs}(x,y) = h_{sim}^{(0\text{cm})}(x,y) + S(x,y) + \epsilon \tag{1}$$

where $S(x,y)$ is the component of error dependent solely on spatial position, and ϵ is the combined sum of all other sources of noise. Assuming that $S(x,y)$ is not sensitive to small changes in global sea surface height, we can improve modeled water elevations by estimating $S(x,y)$ to correct each $h_{sim}^{(X)}(x,y)$. We employ Moving Least Squares (MLS), a mesh-free method of reconstructing functions based on noisy data (8), to reconstruct $S(x,y)$. To do so, we set our MLS support radius to the smallest value r such that all potentially inundated coastal regions between Cape May, New Jersey, and the eastern edge of the Connecticut coast are within distance r to at least one high water observation. After correction, the new modeled water height field, $\tilde{h}_{sim}^{(0\text{cm})}(x,y) = h_{sim}^{(0\text{cm})}(x,y) - S(x,y)$, has a reduced bias of -0.1

cm and RMSE of 22.3 cm, and the error field has no visible spatial correlation remaining (Supplementary Figure 2, “Post-correction” panel).

Spatially-coherent bias on the order of tens of centimeters (see Supplementary Figure 1, Pre-correction panel) is a common result with 2D ocean models that do not capture relatively subtle baroclinic effects such as buoyant coastal trapped flows (e.g. Orton et al. 2012), and methods for incorporating or correcting these errors in 2D models are an active area of research (e.g. (7; 9)). A possible other source for the bias includes erroneous flow through an inlet causing an inaccurate water level across an entire back bay. Further lowering the remaining random errors, which arise for high water marks in upland areas, is a challenge due to the constant evolution and required parameterization of flow over the built landscape (e.g. using a Manning’s-n roughness on a mesh with a minimum resolution of 70 m).

For a few locations, large model vs. observation differences remain even after the correction process (designed not to over-fit the spatial error field). A large difference can arise if there is a wall or tide gate blocking water flow that is not resolved in the model. Alternatively, observational error may be at play. For example, in some locations outside of V-zones (where wave action is more likely to play a role), some high water mark data differ from nearby USGS high water marks by 1 meter even when the data are not marked low quality. Observation error seems likely in these cases. This could arise if there were a debris line that wasn’t a high water mark, for example, due to water runoff.

2 Supplementary Tables

Supplementary Table 1. Estimates of total global mean sea level rise over 1900–2012 in cm. Observed record estimates come from (10) trend estimates over 1900–2012; the observed standard deviation shrinks from 2.6 cm in 1900 to 0.3 cm in 2012, but substantial trend uncertainties remain over the full timeseries (1σ errors of ± 0.4 mm/yr). The budget-based reconstruction is reproduced from Supplementary Table 3. Estimates derived from semi-empirical models are reproduced from Supplementary Table 4 (Historical values, Summary columns) and modified by adding a net global land-water storage (LWS) term (from Supplementary Table 3) for appropriate comparison to observation- and budget-based estimates.

**Source**	*50th*	*(5th–95th)*
Observed record	17.9	(10.5–25.3)
Budget reconstruction	17.5	(12.4–23.1)
Semi-empirical model (HadCRUT4) + LWS	14.9	(9.7–19.2)
Semi-empirical model (CMIP5) + LWS	16.7	(10.3–25.1)

Supplementary Table 2. Estimates of total sea level rise at the Battery, New York over 1900–2012 in cm. For comparison to budget-based sea level reconstructions (reproduced from Supplementary Table 3), vertical land motion from glacial isostatic adjustment (GIA, 1.3 ± 0.4 mm/yr (2σ), from (11), translating to 14.7 cm (10.9–18.5 cm) total) is subtracted from the observed record of total relative sea level rise, 33.8 cm (30.5–37.0 cm). Observed rise is derived by fitting a linear trend over annual mean water levels at NOAA Tide Gauge 8518750 (The Battery, NY, data accessed 29 July 2020); trend uncertainty estimates come directly from NOAA (12). Semi-empirical estimates are derived by scaling global estimates (semi-empirical model values, Supplementary Table 1) by 88% (following the relationship between the global/NY sea-level-rise budgets, each omitting LWS, Supplementary Table 3) and then combining with the NY LWS term median (by addition) and uncertainties (with root sum of squares).

**Source**	*50th*	*(5th–95th)*
Observed record excluding GIA	19.1	(14.1–24.1)
Budget reconstruction	16.7	(11.0–22.8)
Scaled semi-empirical model (HadCRUT4) + NY LWS	14.3	(9.7–18.2)
Scaled semi-empirical model (CMIP5) + NY LWS	16.0	(10.3–23.4)

Supplementary Table 3. Budgets of literature-derived estimated global mean sea level rise, New York area climate-linked sea level rise (plus LWS contributions), and climate-mediated attributable sea level rise, for each over 1900–2012, in cm. Rows of high, central, and low estimates of ASLR reflect ranges of assumed attributable fractions for each sea level component. New York estimates are derived from global ones based on sea level fingerprinting from (13) for each applicable component. Total budgets are derived by summing along each column; component uncertainties are assumed independent and combined with the root sum of squares, i.e. $\sigma_{combined} = \sqrt{\sigma_1^2 + \sigma_2^2 + \cdots + \sigma_n^2}$ on either side of the median assuming piecewise normal distributions. Footnotes detail each term.

**Sea-Level-Rise Budget**			**Attribution** [*High, Central & Low Estimates*]		
Component	*GMSL 50th (5th–95th)*	*New York 50th (5th–95th)*	*% Attribution*	*Global ASLR*[f] *50th (5th–95th)*	*New York ASLR*[f] *50th (5th–95th)*
			69%[c]	1.7 (1.0–2.4)	0.5 (0.3–0.7)
GrIS	2.5 (1.5–3.5)[a]	0.7 (0.4–1.0)[b]	51%[d]	1.3 (0.8–1.8)	0.4 (0.2–0.5)
			32%[e]	0.8 (0.5–1.1)	0.2 (0.1–0.3)
			100%[i]	1.0 (0.7–1.3)	1.4 (0.9–1.8)
AIS	1.0 (0.7–1.3)[g]	1.4 (0.9–1.8)[h]	50%[d]	0.5 (0.3–0.7)	0.7 (0.5–0.9)
			0%[j]	0.0	0.0
			56%[m]	5.0 (3.1–7.0)	4.4 (2.7–6.1)
Glaciers	8.9 (5.4–12.4)[k]	7.8 (4.7–10.8)[ℓ]	40%[m]	3.5 (2.2–4.9)	3.1 (1.9–4.3)
			23%[m]	2.0 (1.2–2.8)	1.8 (1.1–2.5)
			94%[p]	4.9 (1.6–8.8)	4.9 (1.6–8.8)
Thermal expansion	5.2 (1.7–9.4)[n]	5.2 (1.7–9.4)[o]	87%[p]	4.5 (1.5–8.2)	4.5 (1.5–8.2)
			79%[p]	4.1 (1.4–7.4)	4.1 (1.4–7.4)
			100%[r]	—	0.5 (-2.7–3.8)
Ocean dynamics	—	0.5 (-2.7–3.8)[q]	50%[d]	—	0.3 (-1.4–1.9)
			0%[s]	—	0.0

Table 3 continued

**Sea-Level-Rise Budget**			**Attribution** [*High, Central & Low Estimates*]		
Component	*GMSL 50th (5th–95th)*	*New York 50th (5th–95th)*	*% Attribution*	*Global ASLR*[f] *50th (5th–95th)*	*New York ASLR*[f] *50th (5th–95th)*
Land-Water Storage	-0.1 (-0.8–0.5)[t]	1.1 (0.6–1.6)[t]	0%[u]	—	—
Total Budget	17.5 (12.4–23.1)	16.7 (11.0–22.8)		12.6 (8.7–17.1)<br>9.8 (6.4–13.7)<br>7.0 (4.1–10.4)	11.6 (6.7–17.0)<br>8.9 (5.2–13.1)<br>6.1 (3.3–9.5)

[a] Derived with a linear GrIS loss rate over 1900–1971 from (14), and total mass losses over 1972–2012 from (15, their Fig. 3H).
[b] Applying the (13) observed (over 1958–2012) GrIS sea level fingerprint of 0.28 at New York.
[c] Derived assuming GrIS mass losses over 1972–2012 are 100% attributable to anthropogenic forcing, and attribution linearly decreases by 1%/yr from 1971 back to 1900.
[d] Assuming the central estimate is the mean of high and low estimates.
[e] Derived assuming all attributable mass losses are anthropogenic following the emergence of attributable GrIS runoff in 1976 (16), and 0% attribution over 1900–1975.
[f] ASLR computed by multiplying global or New York sea level component contributions by attributable percentages.
[g] Derived as the total AIS mass loss over 1979–2012 from (17, their Fig. 3d). The ice-sheet is assumed to have been net stable over 1900–1978.
[h] Applying the (13) observed (over 1958–2012) AIS sea level fingerprint of 1.35 at New York.
[i] Derived assuming all observed AIS mass losses are anthropogenic. This is an extreme supporting the bounding exercise, but is not necessarily realistic given existing evidence for dominant Antarctic natural variability (see note j).
[j] Natural variability confounds attribution of anthropogenic-driven Antarctic mass loss (e.g. 18, their section 3.3.1.6 and references therein). 0% is derived assuming natural variability dominates mass-loss trends over 1979–2012 (19; 20).
[k] Glacier contribution derived assuming zero contributions over 1900–1901, a linear loss rate over 1902–2005 (21) (their Table 1, as updated from (22)) and over 2006–2012 (23) (their Table 3, based on Table 2 of (24)); GrIS peripheral glaciers are assumed to be net stable over 1900–2012 (21, their Fig. 2). Alternative rates from (25) over 1961–2012, not used here, have a 3.4 cm-lower median when linearly extended back to 1900, but $4\times$ more variance.
[ℓ] Applying the (13) observed (over 1958–2012) glacier sea level fingerprint of 0.88 at New York.
[m] Derived over 1900–2012 by integrating the attributable anthropogenic fractions from 10-year time-slices of $+1\sigma$ (high), median (central), and -1σ (low) 20-year running mean ensembles of (26, their Fig. 3c) with decadal timeseries of Glacier contributions.
[n] Thermal expansion over 1900–2012 derived from bias-corrected modeled 0–700 m depth thermal expansion rates (following 27, their Table 4.1). Bias correction performed with the complementary observed thermal expansion rates over 1970–2012.
[o] Identical to global mean thermal expansion. Deviations are captured by ocean dynamics.
[p] (28) suggest anthropogenic attribution of thermal expansion in the ocean's upper 700 m is $87\pm7\%$ (over 1970–2012; 1σ errors); (29) and (30) (over full depth) agree anthropogenic forcing is strongly consistent with observed thermosteric rise since the 1950s (after which the majority of observed thermosteric SLR took place). We therefore assume $87\pm7\%$ attribution holds over the entire study period.
[q] Derived as the anomaly between thermosteric rise and New York ocean dynamics contributions over 1959–2012 estimated by (31, their Fig. 10) using Subpolar Gyre steric height as a proxy. Loss rates assumed linear over 1964–2012 with steady deceleration backwards to zero over 1959–1963 based on (31, their Table 2), and no net contribution before 1959.
[r] The Subpolar Gyre abruptly warmed in the mid-1990s; assuming all estimated dynamical rise occurred since warming began (based on 31, their Fig. 8b) is anthropogenic, we assume 100% attribution. This is an extreme supporting the bounding exercise, but is not necessarily realistic given existing evidence for dominant North Atlantic natural variability (see Note s).
[s] New York dynamical contributions are strongly correlated with the Subpolar Gyre and hence the strength of the Atlantic meridional overturning circulation (AMOC) (32; 31). But the AMOC has only been continuously observed since the early 2000s (33). Studies of related ocean heat content in this region suggest natural variability dominated over 1950–2000 (e.g. 34). Hence, we assume that a low estimate of attributable ocean-dynamical rise is 0%.
[t] Observed global net change in land-water storage based on dam retention plus groundwater/hydrological contributions (13, their Table 1, and data at New York) over 1958–2012, and no net contribution before 1958.
[u] Assuming land-water storage is not attributable to anthropogenic greenhouse gas forcing.

Supplementary Table 4. Global mean sea level rise estimates for 1900–2012 based on semi-empirical modeling, in cm. Summary results reflect percentiles of simulation results pooled across both temperature calibrations (86,000 and 200,000 samples for CMIP5-based and temperature-scenario-based simulations, respectively). Observed values from (10) included for comparison (see Supplementary Table 1).

			Calibrated to individual temperature reconstructions			
	**Summary**		**Mann et al.**		**Marcott et al.**	
**Scenario**	*50th*	*(5th–95th)*	*50th*	*(5th–95th)*	*50th*	*(5th–95th)*
Observed	17.9	(10.5–25.3)				
CMIP5-based simulations						
Historical	16.8	(10.4–25.2)	19.6	(13.7–26.6)	16.3	(10.1–24.4)
Counterfactual	3.8	(-0.1–9.6)	7.2	(3.3–10.7)	3.1	(-0.2–6.2)
Temperature scenario-based simulations						
Historical*	15.0	(9.8–19.3)	16.1	(13.1–19.6)	13.2	(8.9–18.5)
Stable	4.7	(-0.1–7.8)	6.0	(3.6–8.2)	2.6	(-1.0–6.3)
Cooling	0.7	(-2.9–3.9)	1.0	(-1.5–3.6)	0.3	(-3.7–4.4)
Difference from historical (ASLR)						
CMIP5	12.7	(5.4–21.7)	12.5	(5.4–20.5)	13.3	(5.9–22.4)
Stable	10.4	(7.3–14.3)	10.1	(7.2–13.5)	10.6	(7.3–15.0)
Cooling	14.3	(7.8–20.0)	15.1	(11.4–19.4)	12.9	(6.4–20.7)

Notes: *based on HadCRUT4 temperature record

Supplementary Table 5. Simulated sea level rise from 1900 to 2012 (in cm) for each individual CMIP5 model realization, calibrated to individual temperature reconstructions from Mann et al. and Marcott et al. Summary results reflect percentiles of simulation results pooled across both temperature calibrations. The summary difference column corresponds to estimates of anthropogenic sea level rise (ASLR), the ensemble of which is used for our main calculations and shown in Supplementary Table 4.

	**Summary**						**Mann et al.**						**Marcott et al.**					
	Historical		Counterfactual		Difference		Historical		Counterfactual		Difference		Historical		Counterfactual		Difference	
**Model, Ensemble**	*50th*	*5th−95th*	*50th*	*5th−95th*	*50th*	*5th−95th*	*50th*	*5th−95th*	*50th*	*5th−95th*	*50th*	*5th−95th*	*50th*	*5th−95th*	*50th*	*5th−95th*	*50th*	*5th−95th*
CCSM4, r1i1p1	22.9	(17.8−28.1)	1.6	(-0.2−5.9)	20.1	(16.1−25.6)	24.2	(20.5−28.5)	4.7	(2.9−6.2)	19.5	(16.0−23.6)	21.2	(16.9−27.5)	0.4	(-0.4−1.1)	20.8	(16.2−27.4)
CCSM4, r2i1p1	22.5	(17.5−27.7)	4.0	(2.1−8.1)	17.6	(14.0−22.5)	23.8	(20.1−28.0)	6.8	(5.0−8.5)	17.0	(13.9−20.7)	20.9	(16.5−27.0)	2.5	(2.0−3.5)	18.2	(14.1−24.1)
CCSM4, r4i1p1	23.3	(18.2−28.7)	0.9	(-0.6−5.1)	21.2	(17.0−26.6)	24.7	(20.9−29.1)	4.0	(2.4−5.4)	20.7	(17.1−24.7)	21.6	(17.3−27.8)	-0.1	(-0.8−0.5)	21.7	(17.0−28.3)
CCSM4, r6i1p1	23.4	(18.2−28.7)	1.9	(0.2−6.4)	20.2	(16.2−25.8)	24.7	(20.9−29.1)	5.1	(3.2−6.6)	19.6	(16.1−23.7)	21.7	(17.3−28.1)	0.8	(-0.1−1.6)	20.9	(16.2−27.5)
CNRM-CM5, r1i1p1	15.9	(11.8−19.9)	2.2	(0.7−6.4)	12.6	(10.1−15.8)	17.5	(14.9−20.5)	5.2	(3.6−6.6)	12.3	(10.2−14.6)	14.0	(11.2−17.8)	1.1	(0.7−1.6)	12.9	(10.1−16.8)
CNRM-CM5, r2i1p1	15.7	(11.6−19.6)	5.3	(3.0−9.5)	9.6	(7.5−12.7)	17.2	(14.6−20.2)	8.0	(6.0−9.9)	9.2	(7.5−11.4)	13.9	(11.1−17.8)	3.6	(2.9−4.8)	10.1	(7.7−13.6)
CNRM-CM5, r3i1p1	16.2	(12.0−20.2)	-0.6	(-2.5−3.3)	15.8	(12.8−19.6)	17.8	(15.1−20.9)	2.3	(0.6−3.5)	15.5	(12.8−18.4)	14.3	(11.4−18.2)	-1.8	(-2.7−-1.1)	16.1	(12.7−20.9)
CNRM-CM5, r4i1p1	18.9	(14.4−23.4)	1.7	(0.4−6.0)	15.9	(12.8−20.0)	20.3	(17.3−23.9)	4.9	(3.3−6.3)	15.6	(12.8−18.5)	17.1	(13.7−21.9)	0.8	(0.3−1.3)	16.3	(12.8−21.3)
CNRM-CM5, r5i1p1	14.1	(10.3−17.8)	2.0	(0.4−6.3)	10.9	(8.6−14.1)	15.6	(13.3−18.4)	5.1	(3.3−6.6)	10.5	(8.6−12.9)	12.3	(9.8−15.8)	0.9	(0.2−1.6)	11.3	(8.8−15.1)
CNRM-CM5, r8i1p1	20.6	(15.8−25.4)	0.9	(-0.5−5.1)	18.5	(15.0−22.9)	22.1	(18.8−26.0)	4.0	(2.4−5.3)	18.2	(15.0−21.6)	18.7	(15.0−23.9)	-0.1	(-0.7−0.4)	18.8	(14.9−24.3)
CSIRO-Mk3-6-0, r1i1p1	15.8	(11.6−20.1)	6.8	(4.3−10.3)	8.7	(7.1−10.6)	17.6	(14.9−20.8)	8.9	(7.3−10.7)	8.7	(7.3−10.2)	13.7	(11.1−17.2)	5.1	(4.1−6.3)	8.7	(6.9−10.9)
CSIRO-Mk3-6-0, r2i1p1	13.7	(9.9−17.5)	7.6	(4.9−11.8)	5.5	(4.4−7.4)	15.4	(13.1−18.2)	10.1	(8.1−12.3)	5.3	(4.3−6.6)	11.7	(9.4−14.7)	5.8	(4.6−7.3)	5.9	(4.4−7.9)
CSIRO-Mk3-6-0, r3i1p1	14.2	(10.4−18.0)	3.7	(1.8−7.9)	9.6	(7.6−12.3)	15.8	(13.4−18.6)	6.5	(4.7−8.2)	9.3	(7.6−11.3)	12.3	(9.8−15.6)	2.2	(1.7−3.1)	9.9	(7.7−13.1)
CSIRO-Mk3-6-0, r4i1p1	11.8	(8.3−15.3)	5.2	(3.1−9.3)	5.8	(4.6−7.5)	13.5	(11.4−15.9)	7.9	(6.1−9.7)	5.6	(4.6−6.9)	9.8	(7.9−12.3)	3.7	(2.9−4.7)	6.1	(4.7−8.1)
CSIRO-Mk3-6-0, r5i1p1	16.4	(12.1−20.6)	7.2	(4.6−11.1)	8.7	(7.0−10.9)	18.1	(15.3−21.3)	9.5	(7.7−11.6)	8.6	(7.1−10.2)	14.3	(11.5−18.0)	5.4	(4.3−6.8)	8.9	(7.0−11.6)
CanESM2, r1i1p1	19.1	(14.6−23.6)	2.8	(0.9−7.2)	15.2	(12.0−19.6)	20.5	(17.4−24.2)	5.8	(3.9−7.5)	14.6	(12.0−18.0)	17.4	(13.8−22.5)	1.5	(0.8−2.4)	15.8	(12.2−21.0)
CanESM2, r2i1p1	17.7	(13.4−21.9)	2.4	(0.6−6.7)	14.2	(11.3−18.3)	19.2	(16.3−22.5)	5.4	(3.6−7.0)	13.7	(11.2−16.8)	16.0	(12.7−20.6)	1.1	(0.5−1.9)	14.8	(11.5−19.6)
CanESM2, r3i1p1	18.5	(14.0−22.8)	4.2	(2.0−8.5)	13.4	(10.6−17.4)	19.9	(16.9−23.3)	7.0	(5.0−8.8)	12.8	(10.5−15.9)	16.7	(13.3−21.6)	2.6	(1.8−3.7)	14.0	(10.7−18.6)
CanESM2, r4i1p1	19.9	(15.2−24.5)	4.8	(2.6−9.0)	14.2	(11.3−18.3)	21.3	(18.1−25.0)	7.5	(5.6−9.5)	13.7	(11.2−16.8)	18.1	(14.5−23.2)	3.2	(2.5−4.3)	14.8	(11.4−19.6)
CanESM2, r5i1p1	18.6	(14.2−23.0)	3.7	(1.7−8.1)	13.8	(10.9−18.1)	19.9	(16.9−23.5)	6.6	(4.7−8.4)	13.2	(10.8−16.4)	16.9	(13.4−21.9)	2.3	(1.6−3.3)	14.5	(11.1−19.3)
GFDL-ESM2M, r1i1p1	16.8	(12.5−21.0)	4.9	(2.3−9.3)	11.1	(8.8−14.5)	18.4	(15.6−21.6)	7.7	(5.4−9.8)	10.6	(8.7−13.1)	14.8	(11.9−18.9)	3.1	(2.1−4.6)	11.6	(8.9−15.5)
GISS-E2-H, r1i1p1	23.1	(17.9−28.3)	4.5	(2.3−8.7)	17.8	(14.3−22.2)	24.6	(20.9−29.0)	7.2	(5.3−9.1)	17.4	(14.4−20.7)	21.2	(17.0−27.0)	2.9	(2.2−4.0)	18.2	(14.3−23.7)
GISS-E2-H, r2i1p1	21.5	(16.5−26.4)	6.1	(3.8−10.0)	14.8	(11.9−18.6)	22.9	(19.5−27.0)	8.6	(6.8−10.5)	14.4	(11.9−17.2)	19.7	(15.7−25.0)	4.5	(3.6−5.6)	15.1	(11.8−19.8)
GISS-E2-H, r3i1p1	22.0	(17.0−27.1)	4.8	(2.8−8.9)	16.5	(13.3−20.5)	23.5	(20.0−27.7)	7.5	(5.7−9.3)	16.1	(13.3−19.1)	20.1	(16.1−25.7)	3.3	(2.6−4.2)	16.8	(13.2−21.8)
GISS-E2-H, r4i1p1	20.8	(15.9−25.5)	6.8	(4.3−10.7)	13.4	(10.7−16.9)	22.2	(18.9−26.1)	9.2	(7.4−11.2)	13.0	(10.7−15.6)	19.0	(15.1−24.1)	5.1	(4.1−6.4)	13.7	(10.7−18.1)
GISS-E2-H, r5i1p1	20.6	(15.8−25.3)	4.9	(2.9−9.0)	14.9	(11.9−18.8)	22.0	(18.7−25.9)	7.6	(5.9−9.4)	14.5	(11.9−17.4)	18.8	(15.0−24.0)	3.4	(2.7−4.4)	15.3	(11.9−20.1)
GISS-E2-R, r1i1p1	16.4	(12.2−20.5)	5.0	(2.9−9.1)	10.6	(8.5−13.5)	18.0	(15.3−21.1)	7.7	(5.9−9.5)	10.3	(8.4−12.4)	14.5	(11.6−18.5)	3.5	(2.8−4.5)	11.0	(8.5−14.4)
GISS-E2-R, r2i1p1	18.1	(13.7−22.5)	4.8	(2.6−8.9)	12.5	(10.0−16.0)	19.6	(16.7−23.1)	7.5	(5.5−9.4)	12.1	(9.9−14.7)	16.2	(13.0−20.7)	3.2	(2.4−4.3)	13.0	(10.7−17.1)
GISS-E2-R, r3i1p1	15.4	(11.4−19.3)	2.7	(1.4−7.0)	11.4	(9.2−14.6)	16.9	(14.4−19.9)	5.8	(4.2−7.3)	11.1	(9.1−13.4)	13.6	(10.8−17.5)	1.7	(1.3−2.3)	11.8	(9.2−15.6)
GISS-E2-R, r4i1p1	18.2	(13.8−22.6)	5.9	(3.6−9.8)	11.7	(9.4−14.8)	19.8	(16.8−23.2)	8.4	(6.6−10.2)	11.4	(9.4−13.7)	16.3	(13.1−20.8)	4.2	(3.4−5.3)	12.0	(9.4−15.8)
GISS-E2-R, r5i1p1	18.1	(13.7−22.5)	7.6	(4.9−11.6)	10.0	(7.9−13.0)	19.6	(16.7−23.1)	10.0	(8.0−12.1)	9.6	(7.8−11.9)	16.3	(13.1−20.8)	5.8	(4.6−7.2)	10.5	(8.0−14.0)
HadGEM2-ES, r1i1p1	11.9	(8.4−15.3)	6.3	(3.9−10.3)	4.9	(3.8−6.7)	13.5	(11.5−15.9)	8.8	(7.0−10.8)	4.7	(3.7−6.0)	10.0	(8.0−12.7)	4.6	(3.7−5.8)	5.3	(3.9−7.4)
HadGEM2-ES, r2i1p1	12.5	(8.9−16.1)	7.8	(5.0−12.0)	4.2	(3.2−5.7)	14.2	(12.0−16.8)	10.3	(8.2−12.5)	3.9	(3.1−5.1)	10.5	(8.5−13.2)	6.0	(4.8−7.5)	4.5	(3.3−6.3)
HadGEM2-ES, r3i1p1	13.5	(9.8−17.3)	4.4	(2.3−8.6)	8.3	(6.6−10.8)	15.2	(12.9−17.9)	7.2	(5.2−9.0)	8.0	(6.5−9.9)	11.6	(9.3−14.7)	2.8	(2.1−3.9)	8.7	(6.7−11.5)
HadGEM2-ES, r4i1p1	15.6	(11.5−19.6)	6.0	(3.6−10.1)	8.9	(7.1−11.4)	17.2	(14.6−20.2)	8.6	(6.7−10.6)	8.6	(7.0−10.5)	13.6	(10.9−17.3)	4.3	(3.4−5.5)	9.2	(7.2−12.2)
IPSL-CM5A-LR, r1i1p1	24.7	(19.4−30.3)	5.0	(2.8−9.2)	18.9	(15.1−24.1)	26.1	(22.0−30.8)	7.7	(5.8−9.6)	18.3	(15.1−22.2)	23.1	(18.4−29.6)	3.4	(2.7−4.5)	19.5	(15.2−25.7)
IPSL-CM5A-LR, r2i1p1	24.0	(18.7−29.5)	7.8	(5.1−11.7)	15.6	(12.4−20.1)	25.3	(21.4−29.8)	10.1	(8.2−12.2)	15.1	(12.4−18.5)	22.4	(17.8−28.9)	6.0	(4.8−7.5)	16.3	(12.6−21.6)
IPSL-CM5A-LR, r3i1p1	23.3	(18.2−28.6)	6.4	(3.8−10.7)	16.2	(12.8−20.9)	24.7	(20.9−29.1)	9.1	(6.9−11.2)	15.5	(12.7−19.2)	21.6	(17.3−27.8)	4.6	(3.6−6.1)	16.9	(13.0−22.4)
MIROC-ESM, r1i1p1	18.9	(14.4−23.4)	6.3	(4.0−10.2)	12.0	(9.6−15.1)	20.4	(17.4−24.0)	8.8	(7.1−10.7)	11.7	(9.6−13.9)	17.1	(13.6−21.7)	4.7	(3.8−5.9)	12.3	(9.6−16.0)
MIROC-ESM-CHEM, r1i1p1	18.4	(13.9−22.8)	7.1	(4.5−11.1)	10.7	(8.5−13.8)	19.9	(16.9−23.4)	9.5	(7.6−11.6)	10.3	(8.5−12.7)	16.5	(13.2−21.1)	5.3	(4.2−6.6)	11.2	(8.6−14.8)
MRI-CGCM3, r1i1p1	12.9	(9.2−16.6)	2.9	(1.1−7.2)	9.0	(7.2−11.4)	14.6	(12.4−17.2)	5.8	(4.0−7.5)	8.8	(7.2−10.5)	10.9	(8.8−13.7)	1.6	(1.0−2.4)	9.3	(7.2−12.1)
NorESM1-M, r1i1p1	13.7	(10.0−17.4)	4.9	(2.8−9.0)	8.0	(6.3−10.6)	15.4	(13.1−18.1)	7.6	(5.7−9.5)	7.7	(6.3−9.6)	11.9	(9.5−15.2)	3.3	(2.6−4.4)	8.4	(6.4−11.3)
bcc-csm1-1, r1i1p1	19.7	(15.2−24.3)	5.3	(3.2−9.4)	13.6	(10.7−17.8)	21.1	(17.8−24.8)	7.9	(6.2−9.8)	13.0	(10.6−16.1)	18.1	(14.3−23.5)	3.8	(3.0−4.8)	14.2	(10.9−19.0)

Supplementary Table 6. Percentage of property damage, flood volume, and population and housing exposure attributable to different estimates of anthropogenic sea level rise (ASLR) under various model variations. Models components include hydrodynamic simulation (S), spatial bias-correction (BC), and flood-depth-based modeling of property damage (D), whereby our main model consists of S+BC+D. Percentage of damage can be approximated using flood volume ratios, without the need for damage models (see rows labeled "Damage proxy"). Estimates from both the non-bias-corrected model (S+D) and the flood volume-based model (S) serve as reasonable alternatives, with relative errors of -4.2% and 9.9%, respectively. These errors were calculated using the ratio of the percentage of attributable impact under the S+D or S models, respectively, relative to percentage of attributable damage under the full S+BC+D model, all using 50th percentiles from the total ensemble ASLR estimate as listed in the table.

		**Percentage attributable to ASLR**		
	**Model**	**Total ensemble** *50th (5th–95th)*	**Budget-based** *50th (5th–95th)*	**Semi-empirical ensemble** *50th (5th–95th)*
Tri-State Area				
Damage	S+BC+D	14.2% (8.9%–24.4%)	13.3% (8.6%–19.7%)	16.0% (9.8%–26.8%)
Damage	S+D	13.6% (8.5%–23.6%)	12.7% (8.3%–19.0%)	15.4% (9.4%–26.0%)
Damage Proxy	S	15.6% (10.1%–25.1%)	14.7% (9.8%–20.7%)	17.4% (11.1%–27.3%)
Population	S+BC+D	10.1% (6.3%–18.7%)	9.4% (6.1%–14.8%)	11.7% (6.9%–20.8%)
Population	S+D	9.9% (6.3%–18.2%)	9.2% (6.1%–14.4%)	11.4% (6.9%–20.2%)
Housing	S+BC+D	9.7% (5.9%–17.5%)	9.0% (5.7%–13.9%)	11.2% (6.6%–19.5%)
Housing	S+D	9.5% (5.9%–17.0%)	8.8% (5.7%–13.5%)	10.9% (6.6%–18.9%)
New York				
Damage	S+BC+D	14.5% (9.1%–23.2%)	13.5% (8.8%–18.5%)	16.4% (10.0%–25.7%)
Damage	S+D	14.8% (9.3%–23.7%)	13.8% (9.0%–18.9%)	16.8% (10.3%–26.4%)
Damage Proxy	S	17.3% (11.1%–26.6%)	16.3% (10.8%–21.8%)	19.4% (12.3%–29.2%)
Population	S+BC+D	10.5% (6.5%–17.6%)	9.7% (6.2%–13.6%)	12.3% (7.2%–19.9%)
Population	S+D	10.7% (6.7%–17.6%)	9.9% (6.5%–13.7%)	12.4% (7.4%–19.9%)
Housing	S+BC+D	10.2% (6.2%–17.1%)	9.4% (6.0%–13.2%)	11.9% (6.9%–19.6%)
Housing	S+D	10.3% (6.5%–17.1%)	9.6% (6.3%–13.3%)	11.9% (7.2%–19.5%)
New Jersey				
Damage	S+BC+D	14.0% (8.8%–26.1%)	13.1% (8.5%–21.3%)	15.7% (9.7%–28.4%)
Damage	S+D	12.4% (7.8%–23.4%)	11.7% (7.5%–19.1%)	14.0% (8.6%–25.5%)
Damage Proxy	S	14.3% (9.4%–24.1%)	13.5% (9.2%–20.1%)	15.9% (10.3%–26.0%)
Population	S+BC+D	9.6% (6.0%–21.0%)	8.9% (5.8%–16.9%)	10.9% (6.7%–22.8%)
Population	S+D	8.7% (5.5%–19.3%)	8.2% (5.4%–15.6%)	9.9% (6.2%–21.0%)
Housing	S+BC+D	9.3% (5.7%–18.1%)	8.6% (5.5%–14.7%)	10.7% (6.3%–19.7%)
Housing	S+D	8.6% (5.3%–16.9%)	8.0% (5.2%–13.8%)	9.8% (5.9%–18.3%)
Connecticut				
Damage	S+BC+D	11.4% (7.1%–18.8%)	10.7% (6.9%–15.1%)	13.1% (7.9%–20.9%)
Damage	S+D	13.2% (7.8%–22.0%)	12.3% (7.5%–17.8%)	15.0% (8.6%–24.4%)
Damage Proxy	S	14.0% (8.2%–22.0%)	13.1% (8.0%–18.1%)	15.7% (9.1%–24.1%)
Population	S+BC+D	7.5% (4.6%–12.6%)	6.9% (4.4%–10.1%)	8.9% (5.1%–14.1%)
Population	S+D	8.7% (5.1%–14.5%)	8.1% (4.9%–11.8%)	9.9% (5.6%–16.1%)
Housing	S+BC+D	8.1% (4.8%–13.7%)	7.4% (4.7%–10.9%)	9.7% (5.4%–15.2%)
Housing	S+D	9.4% (5.5%–15.6%)	8.7% (5.3%–12.7%)	10.8% (6.1%–17.3%)

3 Supplementary Figures

Supplementary Figure 1. Sandy modeled (shading) vs. observed (dots) maximum water elevations relative to NAVD88 along the Atlantic coast of New Jersey, New York, and Connecticut.

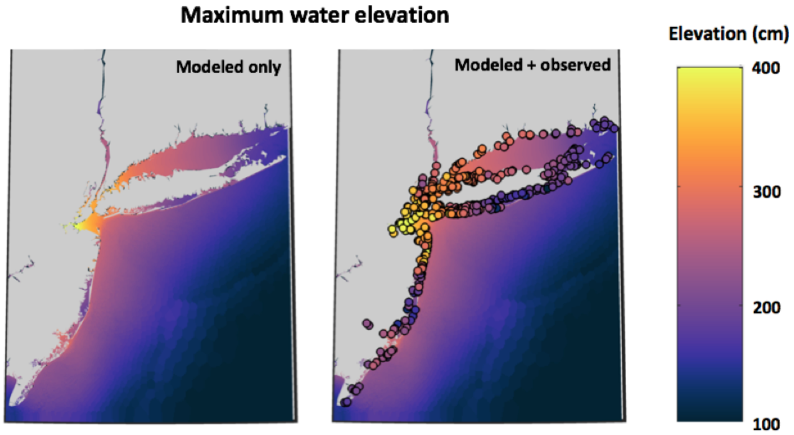

Supplementary Figure 2. Residuals in cm of modeled vs. observed high water levels from Sandy, without and then with spatially variable error correction.

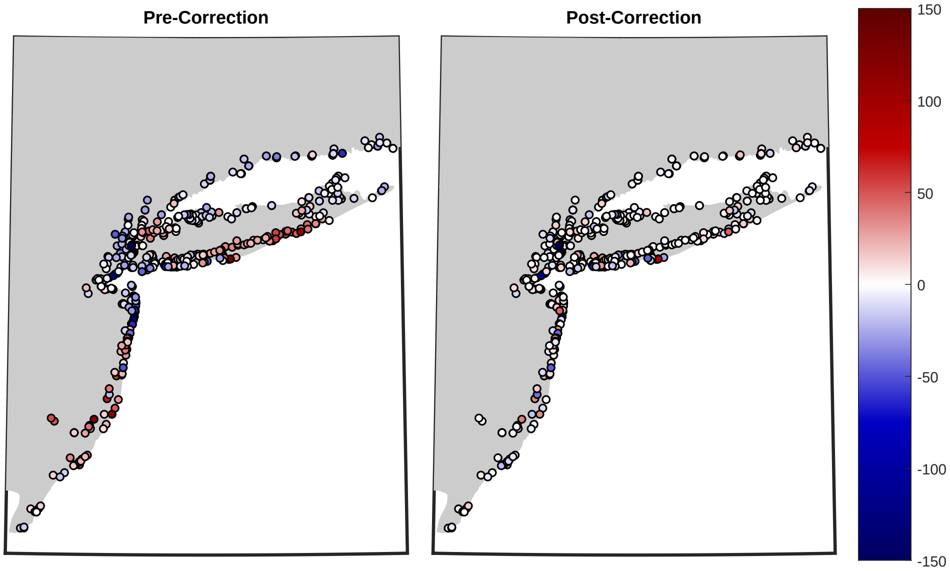

Supplementary Figure 3. County-level estimates for percentage of damage attributable to anthropogenic SLR using median (5th-95th percentile) ASLR under total ensemble attribution of sea level rise and a full model including storm surge, bias correction, and damage. Counties color-coded by state: blue for Connecticut, magenta for New York, and red for New Jersey. The wide interval for Monmouth County, NJ is due in large part to a waterfront sand dune system and inland levees surrounding three sides of Keansburg, which flood waters defeated in the actual event, but which modeling indicates would have provided substantial protection in counterfactual scenarios incorporating large amounts of ASLR (e.g. the 95th percentile case). The wide interval for Union County appears to come from several densely developed Census blocks which transition between flooded and dry within the ASLR range mapped.

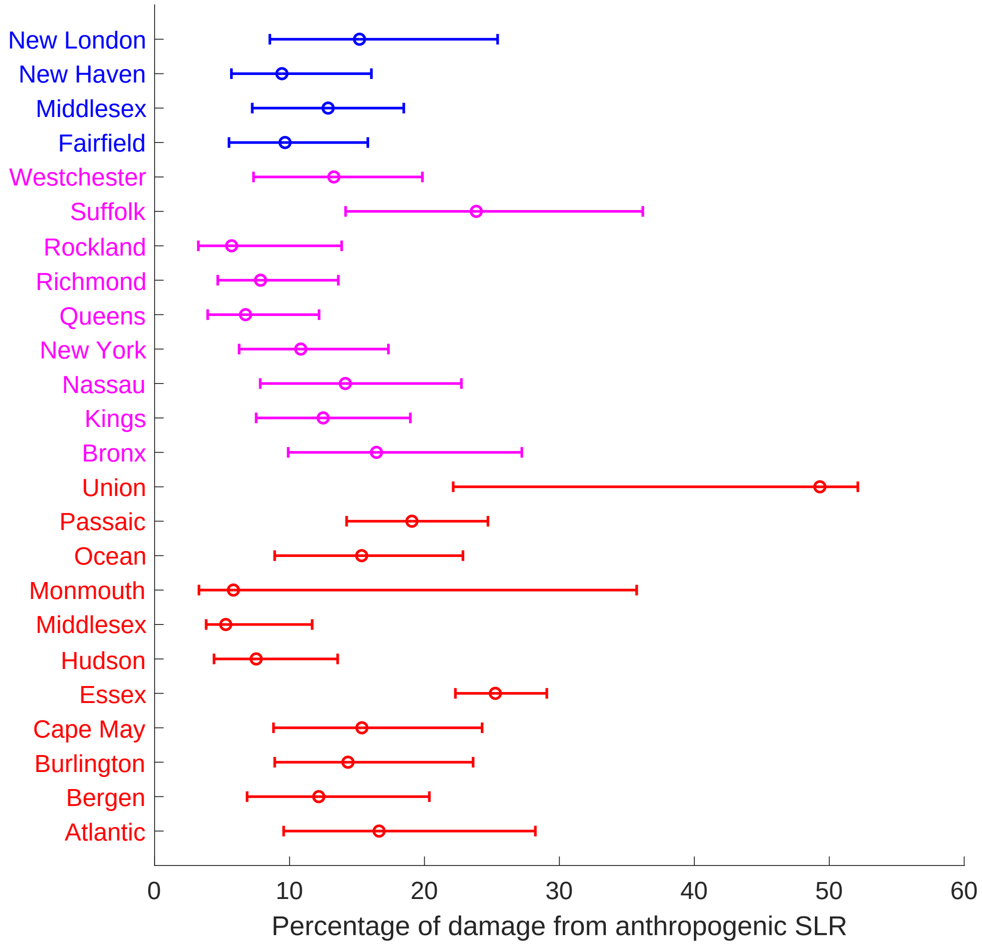

Supplementary Figure 4. Model comparisons. (A) Percentage of damage attributable to anthropogenic sea level rise (ASLR). Simulated and bias-corrected compared to non-bias-corrected values at the county level. One-to-one line displayed in black. (B) Percentage of damage and flood volume attributable to ASLR. Simulated percentage of damage vs. percentage of flood volume at the county level.

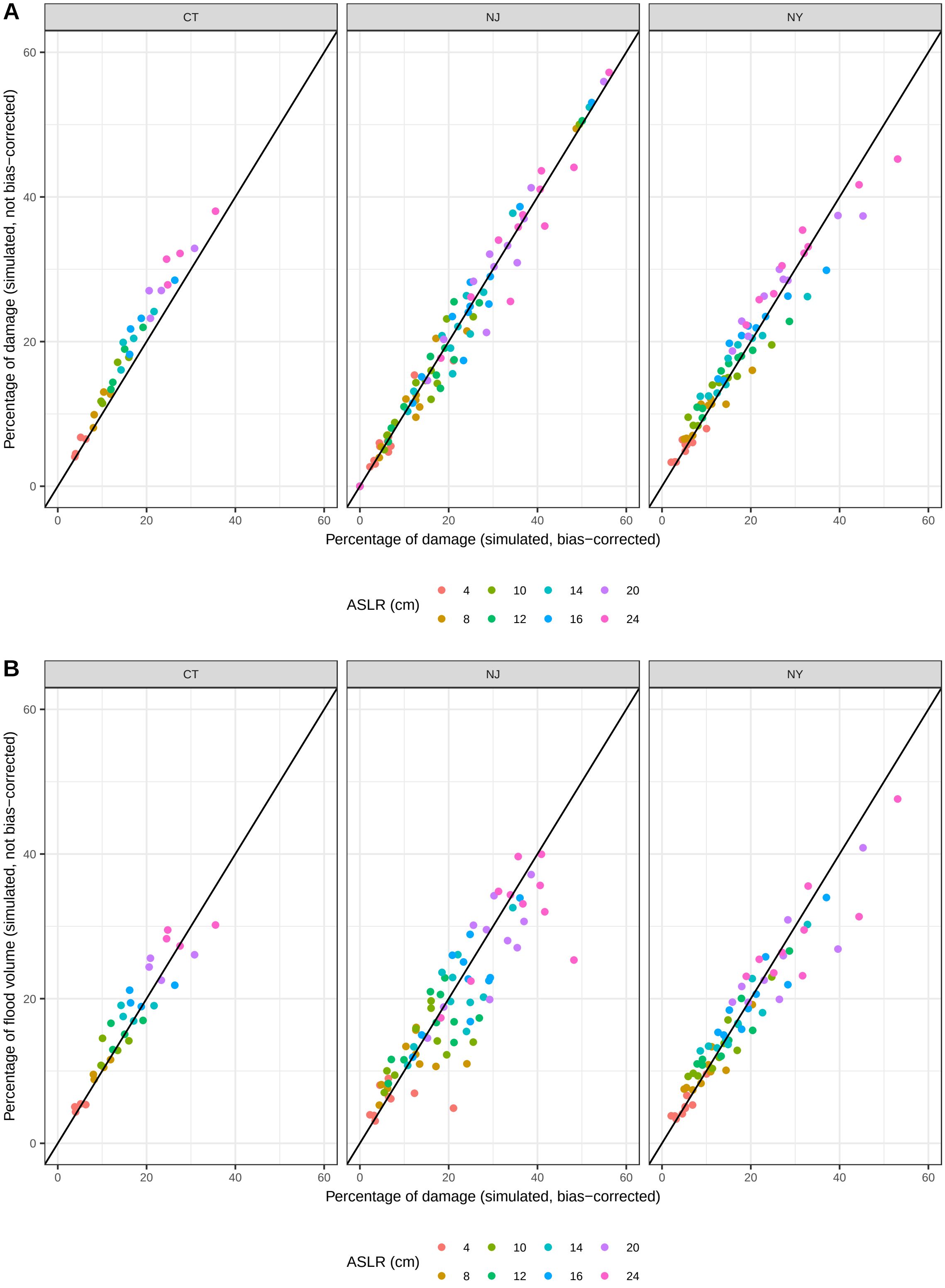

4 Supplementary References

Bibliography

[1] M. A. Cialone, T. C. Massey, M. E. Anderson, A. S. Grzegorzewski, R. E. Jensen, A. Cialone, D. J. Mark, K. C. Pevey, B. L. Gunkel, and T. O. McAlpin, "North atlantic coast comprehensive study (naccs) coastal storm model simulations: Waves and water levels," tech. rep., Engineer Research and Development Center, 2015.

[2] W. D. Federal Emergency Management Agency, US Department of Homeland Security, "Region ii storm surge project mesh development," 2014.

[3] T. C. Massey, M. E. Anderson, J. M. Smith, J. Gomez, and R. Jones, "Stwave: Steady-state spectral wave model. users manual for stwave, version 6.0.," tech. rep., ERDC/CHL SR-11-1, U.S. Army Engineer Research and Development Center, Vicksburg, MS, 2011.

[4] J. Garratt, "Review of drag coefficients over oceans and continents," *Monthly weather review*, vol. 105, no. 7, pp. 915–929, 1977.

[5] Federal Emergency Management Agency, "Redefinition of the coastal flood hazard zones in fema region ii: Analysis of the coastal storm surge flood frequencies," tech. rep., Federal Emergency Management Agency, 2014.

[6] P. Orton, N. Lin, V. Gornitz, B. Colle, J. Booth, K. Feng, M. Buchanan, M. Oppenheimer, and L. Patrick, "New york city panel on climate change 2019 report chapter 4: coastal flooding," *Annals of the New York Academy of Sciences*, vol. 1439, no. 1, pp. 95–114, 2019.

[7] A. Thomas, J. Dietrich, T. Asher, M. Bell, B. Blanton, J. Copeland, A. Cox, C. Dawson, J. Fleming, and R. Luettich, "Influence of storm timing and forward speed on tides and storm surge during hurricane matthew," *Ocean Modelling*, vol. 137, pp. 1–19, 2019.

[8] D. Levin, "The approximation power of moving least-squares," *Mathematics of Computation*, vol. 67, pp. 1517–1532, oct 1998.

[9] T. G. Asher, R. A. Luettich Jr, J. G. Fleming, and B. O. Blanton, "Low frequency water level correction in storm surge models using data assimilation," *Ocean Modelling*, vol. 144, p. 101483, 2019.

[10] S. Dangendorf, C. Hay, F. Calafat, M. Marcos, C. Piecuch, K. Berk, and J. Jensen, "Persistent acceleration in global sea-level rise since the 1960s," *Nature Climate Change*, vol. 9, 09 2019.

[11] R. E. Kopp, "Does the mid-atlantic united states sea level acceleration hot spot reflect ocean dynamic variability?," *Geophysical Research Letters*, vol. 40, no. 15, pp. 3981–3985, 2013.

[12] C. Zervas, "Sea Level Variations of the United States 1854-2006," *Technical Report NOS CO-OPS 053*, vol. 53, no. December, 2009.

[13] T. Frederikse, R. Riva, M. Kleinherenbrink, Y. Wada, M. van den Broeke, and B. Marzeion, "Closing the sea level budget on a regional scale: Trends and variability on the Northwestern European continental shelf," *Geophysical Research Letters*, vol. 43, no. 20, pp. 10,864–10,872, 2016.

[14] K. K. Kjeldsen, N. J. Korsgaard, A. A. Bjørk, S. A. Khan, J. E. Box, S. Funder, N. K. Larsen, J. L. Bamber, W. Colgan, M. Van Den Broeke, M. L. Siggaard-Andersen, C. Nuth, A. Schomacker, C. S. Andresen, E. Willerslev, and K. H. Kjær, "Spatial and temporal distribution of mass loss from the Greenland Ice Sheet since AD 1900," *Nature*, vol. 528, no. 7582, pp. 396–400, 2015.

[15] J. Mouginot, E. Rignot, A. A. Bjørk, M. van den Broeke, R. Millan, M. Morlighem, B. Noël, B. Scheuchl, and M. Wood, "Forty-six years of Greenland Ice Sheet mass balance from 1972 to 2018," *Proceedings of the National Academy of Sciences of the United States of America*, vol. 116, no. 19, pp. 9239–9244, 2019.

[16] L. D. Trusel, S. B. Das, M. B. Osman, M. J. Evans, B. E. Smith, X. Fettweis, J. R. McConnell, B. P. Noël, and M. R. van den Broeke, "Nonlinear rise in Greenland runoff in response to post-industrial Arctic warming," *Nature*, vol. 564, no. 7734, pp. 104–108, 2018.

[17] E. Rignot, J. Mouginot, M. van den Broeke, M. J. van Wessem, M. Morlighem, and B. Scheuchl, "Four decades of Antarctic Ice Sheet mass balance from 19792017," *Proceedings of the National Academy of Sciences*, vol. 116, no. 4, pp. 1095–1103, 2019.

[18] M. S. S. C. C. D. A. E. A. H. G. K. A. M. J. M.-T. M. M. G. O. H. P. Meredith, M. and E. A. G. Schuur, "Polar regions.," 2019.

[19] K. L. Smith and L. M. Polvani, "Spatial patterns of recent Antarctic surface temperature trends and the importance of natural variability: lessons from multiple reconstructions and the CMIP5 models," *Climate Dynamics*, vol. 48, no. 7, pp. 2653–2670, 2017.

[20] A. Jenkins, D. Shoosmith, P. Dutrieux, S. Jacobs, T. W. Kim, S. H. Lee, H. K. Ha, and S. Stammerjohn, "West Antarctic Ice Sheet retreat in the Amundsen Sea driven by decadal oceanic variability," *Nature Geoscience*, vol. 11, no. 10, pp. 733–738, 2018.

[21] B. Marzeion, P. W. Leclercq, J. G. Cogley, and A. H. Jarosch, "Brief Communication: Global reconstructions of glacier mass change during the 20th century are consistent," *Cryosphere*, vol. 9, no. 6, pp. 2399–2404, 2015.

[22] B. Marzeion, A. H. Jarosch, and M. Hofer, "Past and future sea-level change from the surface mass balance of glaciers," *Cryosphere*, vol. 6, no. 6, pp. 1295–1322, 2012.

[23] G. S. L. B. G. WCRP, "Global sea-level budget 1993-present," *Earth System Science Data*, vol. 10, no. 3, pp. 1551–1590, 2018.

[24] B. Marzeion, N. Champollion, W. Haeberli, K. Langley, P. Leclercq, and F. Paul, "Observation-Based Estimates of Global Glacier Mass Change and Its Contribution to Sea-Level Change," *Surveys in Geophysics*, vol. 38, no. 1, pp. 105–130, 2017.

[25] M. Zemp, M. Huss, E. Thibert, N. Eckert, R. McNabb, J. Huber, M. Barandun, H. Machguth, S. U. Nussbaumer, I. Gärtner-Roer, L. Thomson, F. Paul, F. Maussion, S. Kutuzov, and J. G. Cogley, "Global glacier mass changes and their contributions to sea-level rise from 1961 to 2016," *Nature*, vol. 568, no. 7752, pp. 382–386, 2019.

[26] M. Marcos, B. Marzeion, S. Dangendorf, A. B. Slangen, H. Palanisamy, and L. Fenoglio-Marc, "Internal variability versus anthropogenic forcing on sea level and its components," *Surveys in Geophysics*, vol. 38, no. 1, pp. 329–348, 2017.

[27] M. Oppenheimer, B. Glavovic, J. Hinkel, R. van de Wal, A. K. Magnan, A. Abd-Elgawad, R. Cai, M. Cifuentes-Jara, R. M. Deconto, T. Ghosh, J. Hay, F. Isla, B. Marzeion, B. Meyssignac, and Z. Sebesvari, "Sea level rise and implications for low lying islands, coasts and communities," 2019.

[28] M. Marcos and A. Amores, "Quantifying anthropogenic and natural contributions to thermosteric sea level rise," *Geophysical Research Letters*, vol. 41, no. 7, pp. 2502–2507, 2014.

[29] A. B. Slangen, J. A. Church, X. Zhang, and D. Monselesan, "Detection and attribution of global mean thermosteric sea level change," *Geophysical Research Letters*, vol. 41, no. 16, pp. 5951–5959, 2014.

[30] K. B. Tokarska, G. C. Hegerl, A. P. Schurer, A. Ribes, and J. T. Fasullo, "Quantifying human contributions to past and future ocean warming and thermosteric sea level rise," *Environmental Research Letters*, vol. 14, no. 7, p. 074020, 2019.

[31] T. Frederikse, K. Simon, C. A. Katsman, and R. Riva, "The sea-level budget along the northwest atlantic coast: Gia, mass changes, and large-scale ocean dynamics," *Journal of Geophysical Research: Oceans*, vol. 122, no. 7, pp. 5486–5501, 2017.

[32] J. Yin and P. B. Goddard, "Oceanic control of sea level rise patterns along the East Coast of the United States," *Geophysical Research Letters*, vol. 40, no. 20, pp. 5514–5520, 2013.

[33] E. Frajka-Williams, I. J. Ansorge, J. Baehr, H. L. Bryden, M. P. Chidichimo, S. A. Cunningham, G. Danabasoglu, S. Dong, K. A. Donohue, S. Elipot, P. Heimbach, N. P. Holliday, R. Hummels, L. C. Jackson, J. Karstensen, M. Lankhorst, I. A. Le Bras, M. Susan Lozier, E. L. McDonagh, C. S. Meinen, H. Mercier, B. I. Moat, R. C. Perez, C. G. Piecuch, M. Rhein, M. A. Srokosz, K. E. Trenberth, S. Bacon, G. Forget, G. Goni, D. Kieke, J. Koelling, T. Lamont, G. D. McCarthy, C. Mertens, U. Send, D. A. Smeed, S. Speich, M. van den Berg, D. Volkov, and C. Wilson, "Atlantic meridional overturning circulation: Observed transport and variability," *Frontiers in Marine Science*, vol. 6, no. JUN, pp. 1–18, 2019.

[34] M. S. Lozier, S. Leadbetter, R. G. Williams, V. Roussenov, M. S. Reed, and N. J. Moore, "The spatial pattern and mechanisms of heat-content change in the North Atlantic," *Science*, vol. 319, no. 5864, pp. 800–803, 2008.
