# Supplementary material for: Economic damages from Hurricane Sandy attributable to sea level rise caused by anthropogenic climate change
Source: Nat Commun. 2021 May 18;12:2720. doi: 10.1038/s41467-021-22838-1 (PMC8131618; doi:10.1038/s41467-021-22838-1)
Supplement: Supplementary file 3 — Reporting Summary [file 41467_2021_22838_MOESM3_ESM.pdf]

nature research

Corresponding author(s): Benjamin Strauss

Last updated by author(s): Mar 10, 2021

Reporting Summary

Nature Research wishes to improve the reproducibility of the work that we publish. This form provides structure for consistency and transparency in reporting. For further information on Nature Research policies, see our Editorial Policies and the Editorial Policy Checklist.

Statistics

For all statistical analyses, confirm that the following items are present in the figure legend, table legend, main text, or Methods section.

n/a	Confirmed	
☒	☐	The exact sample size (n) for each experimental group/condition, given as a discrete number and unit of measurement
☒	☐	A statement on whether measurements were taken from distinct samples or whether the same sample was measured repeatedly
☒	☐	The statistical test(s) used AND whether they are one- or two-sided<br>*Only common tests should be described solely by name; describe more complex techniques in the Methods section.*
☒	☐	A description of all covariates tested
☐	☒	A description of any assumptions or corrections, such as tests of normality and adjustment for multiple comparisons
☐	☒	A full description of the statistical parameters including central tendency (e.g. means) or other basic estimates (e.g. regression coefficient) AND variation (e.g. standard deviation) or associated estimates of uncertainty (e.g. confidence intervals)
☒	☐	For null hypothesis testing, the test statistic (e.g. F, t, r) with confidence intervals, effect sizes, degrees of freedom and P value noted<br>*Give P values as exact values whenever suitable.*
☒	☐	For Bayesian analysis, information on the choice of priors and Markov chain Monte Carlo settings
☒	☐	For hierarchical and complex designs, identification of the appropriate level for tests and full reporting of outcomes
☒	☐	Estimates of effect sizes (e.g. Cohen's d, Pearson's r), indicating how they were calculated

Our web collection on statistics for biologists contains articles on many of the points above.

Software and code

Policy information about availability of computer code

Data collection	No software was used for data collection in this study.
Data analysis	Hydrodynamic simulation was all performed with publicly available software: ADCIRC version 52.30, STWAVE version 6.2.28, and CSTORM Coupling Software version 1.1.16. Custom Matlab code (R2017b+R2018b) for performing spatial bias corrections, semi-empirical analyses, and observational analyses are documented and publicly available for download at https://github.com/climatecentral/cc_sandy_matlab. Methods related to assessing land, population, and housing exposure were implemented using custom Matlab (R2017b), Python, and C++ code. Due to licensing restrictions by Climate Central, this code is not publicly available. However, these tasks can be accomplished with standard GIS software. Damage assessment was performed using HAZUS-MH software version MR4.

For manuscripts utilizing custom algorithms or software that are central to the research but not yet described in published literature, software must be made available to editors and reviewers. We strongly encourage code deposition in a community repository (e.g. GitHub). See the Nature Research guidelines for submitting code & software for further information.

Data

Policy information about availability of data

All manuscripts must include a data availability statement. This statement should provide the following information, where applicable:

- Accession codes, unique identifiers, or web links for publicly available datasets
- A list of figures that have associated raw data
- A description of any restrictions on data availability

Input/output/source data files are archived at doi.org/10.5281/zenodo.4302772, including source data files for all figures and tables in the manuscript and in supplementary information.

Publicly available datasets used as direct inputs are available as follows:

* HadCRUT4 annual mean global temperature reconstructions from https://www.metoffice.gov.uk/hadobs/hadcrut4/data/current/download.html;
* CMIP5 global temperature simulations from https://esgf-node.llnl.gov/projects/cmip5/;
parameter distributions for the semi-empirical sea level model, dataset S01(j) from https://www.pnas.org/content/suppl/2016/02/17/1517056113.DCSupplemental;
* global mean sea level timeseries reconstruction from Dangendorf et al (2019) supplementary data 1, https://static-content.springer.com/esm/art%3A10.1038%2Fs41558-019-0531-8/MediaObjects/41558_2019_531_MOESM2_ESM.txt;
* New York City sea level observations from https://tidesandcurrents.noaa.gov/sltrends/sltrends_station.shtml?id=8518750;
* tide gauge uncertainty estimates from NOAA Technical Report NOS CO-OPS 053 53, Figure 30, https://tidesandcurrents.noaa.gov/publications/Tech_rpt_53.pdf;
* glacial isostatic adjustment estimates from spatio-temporal modeling of Kopp (2013) from supporting information Table S1, https://agupubs.onlinelibrary.wiley.com/action/downloadSupplement?doi=10.1002%2Fgrl.50781&file=Kopp2013_SI.pdf; and
* USGS data on Sandy high water levels from https://doi.org/10.3133/sir20155036 and https://doi.org/10.3133/sir20165085.

Field-specific reporting

Please select the one below that is the best fit for your research. If you are not sure, read the appropriate sections before making your selection.

☐ Life sciences ☐ Behavioural & social sciences ☒ Ecological, evolutionary & environmental sciences

For a reference copy of the document with all sections, see nature.com/documents/nr-reporting-summary-flat.pdf

Ecological, evolutionary & environmental sciences study design

All studies must disclose on these points even when the disclosure is negative.

Study description

This study compares modeled damages from Hurricane Sandy as it occurred and as it could have occurred were sea level lower. It uses simulation of flooding and damages based on a small set of different baseline sea levels. Starting sea level is the only independent variable distinguishing the simulations.

Separate analyses characterize the distribution of New-York-area sea level rise between 1900 and 2012 (when Sandy occurred) attributable to human-caused climate change. A budget-based approach relies on compiling existing literature. A second approach reconstructs sea levels with and without anthropogenic climate change by applying a semi-empirical sea level model based on global temperature. This approach involves sampling from distributions of the model's parameters (under each of two different model calibrations) and from reconstructed-historical or counterfactual temperatures.

Research sample

This study employs multiple existing large datasets as referenced in the Data section above and described in the manuscript.

The parameter distributions for the semi-empirical sea level model come from Kopp et al (2016), PNAS, and are accessible in dataset S01 (j) in the supplementary material of that paper.

For historical and counterfactual mean global temperature timeseries, the study used all 43 fully applicable CMIP5 realizations, extended linearly from 2005 through 2012; the HadCRUT4 historical global mean annual temperature reconstruction; and 100 counterfactual temperature timeseries per semi-empirical model parameter combination.

Historical New York monthly mean sea level observations from a tide gauge were used to help validate sea level reconstructions; trends were corrected to account for glacial isostatic adjustment with the GIA estimates constructed in Kopp (2013). Historical tide gauge uncertainties were derived from NOAA's technical assessment.

The study used USGS data from sensors and field observations on Sandy peak water levels. All data in the study area rated better than poor were included.

Sampling strategy

The calibrated semi-empirical sea-level model of Kopp et al. (2016) was used to produce global mean sea level estimates in 2012 (relative to 1900) as a function of global mean temperature, driven by historical or counterfactual scenarios.

Temperature-based scenario simulations, assuming stable or cooling counterfactual temperatures or historical HadCRUT4 temperatures, have distributions formed by pooling across 2(model calibrations) x 1,000(parameter sets) x 100(temperature timeseries)=200,000 samples per scenario.

This study used the exact calibrations of Kopp et al. (2016); a brief description of the approach is as follows. The semi-empirical model was calibrated by fitting output driven by two common-era temperature reconstructions (Mann et al. 2009 and Marcott et al. 2013) against the global mean sea level reconstruction of Kopp et al. (2016). Each of the two separate resulting calibrations (hereafter, Marcott and Mann) were composed of 1,000 parametric sets which maximized the likelihood that model output reproduced the target common-era global mean sea levels.

For each of the stable counterfactual, cooling counterfactual, and historical HadCRUT4 scenarios, 100 global-mean temperature timeseries (over 1900-2012) were drawn as normally distributed observations with AR(1) noise deviating from the central timeseries values defined by each scenario, following the approach described in Kopp et al. 2016. For each of these generated timeseries, the full range of semi-empirical model outputs were produced from the 1,000 parameter sets, ultimately resulting in 100,000 samples of

global-mean sea level increases across each of the Marcott and Mann calibrations (a total of 200,000 samples pooling across both calibrations).

CMIP5-based simulations, assuming either modeled historical or counterfactual temperature scenarios, have distributions pooled across 2(model calibrations) x 1,000(parameters sets) x 43(CMIP5 model realizations)=86,000 samples per scenario. A global-mean temperature timeseries was drawn from each of the 43 model realizations of modeled historical and counterfactual scenarios. As above, the full range of semi-empirical model outputs was produced with each modeled-temperature timeseries and the 1,000 parameter sets, ultimately resulting in 43,000 samples of global-mean sea level increases across each of the Marcott and Mann calibrations (a total of 86,000 samples pooling across both calibrations).

For each of the three counterfactual scenarios (stable, cooling, or CMIP5), a distribution of attributable sea level rise was developed from paired sample differences with the corresponding historical scenarios. Each distribution was then sampled equally 100 million times, with replacement, to develop an overall pooled semi-empirical ensemble of 300 million samples of attributable sea level rise in total.

This study also developed a budget-based distribution of attributable sea level rise requiring sampling and combination with the semi-empirical model ensemble. Before sampling, skew-normal distributions were fit by minimizing the root-mean square error between the known percentiles and estimated percentiles from 500,000 randomly generated skew-normal distributions.

The integrated summary (total ensemble) of attributable sea level rise was formed by equally weighting the budget-based and semi-empirical model approaches. The optimal-fit budget-based distribution of attributable sea level rise was sampled 300 million times to match the number of samples from the semi-empirical model ensemble. Pooling across both methods formed the total ensemble distribution made from the combined 600 million samples.

Where applicable, the same procedures were separately performed for distributions of global and New York-area attributable sea level rise.

Data collection

No new data were collected.

Timing and spatial scale

No new data were collected.

Data exclusions

USGS high water level data rated as poor by the USGS were excluded from use in our assessment of our simulation of Sandy's flood.

GFDL-CM3 model runs were excluded from the CMIP5 ensemble analysis due to a historical data discontinuity over 1960-1980 (after this exclusion, 43 CMIP5 model realizations remained).

Reproducibility

No replication was attempted. The very large sample sizes and deterministic approaches employed support a high degree of reproducibility, as do the code archives, data archives, data citations, and methodological description provided.

Randomization

Not applicable for the main analysis because only one flood simulation was run per sea level tested, and sea level was the only independent variable. Sampling of inputs to develop distributions of sea level rise attributable to human-caused climate change is described under Sampling strategy, above.

Blinding

Not applicable as results do not depend on subjective scoring.

Did the study involve field work? ☐ Yes ☒ No

Reporting for specific materials, systems and methods

We require information from authors about some types of materials, experimental systems and methods used in many studies. Here, indicate whether each material, system or method listed is relevant to your study. If you are not sure if a list item applies to your research, read the appropriate section before selecting a response.

Materials & experimental systems

n/a	Involved in the study	
☒	☐	Antibodies
☒	☐	Eukaryotic cell lines
☒	☐	Palaeontology and archaeology
☒	☐	Animals and other organisms
☒	☐	Human research participants
☒	☐	Clinical data
☒	☐	Dual use research of concern

Methods

n/a	Involved in the study	
☒	☐	ChIP-seq
☒	☐	Flow cytometry
☒	☐	MRI-based neuroimaging
